# Enhancement of Stability and Antioxidant Activity of Mulberry Anthocyanins Through Succinic Acid Acylation

**DOI:** 10.17113/ftb.60.03.22.7203

**Published:** 2022-09

**Authors:** Bei Zhang, Xizhi Jiang, Gaiqun Huang, Xiangdong Xin, Thomas Attaribo, Yueyue Zhang, Ning Zhang, Zhongzheng Gui

**Affiliations:** 1 School of Biotechnology, Jiangsu University of Science and Technology, #666 Changhui Road, Zhenjiang, Jiangsu 212018, PR China; 2 Sericultural Research Institute, Sichuan Academy of Agricultural Sciences, #87 Hezhong Street, Nanchong, Sichuan 637000, PR China; 3 Sericultural Research Institute, Chinese Academy of Agricultural Sciences, #666 Changhui Road, Zhenjiang, Jiangsu 212018, PR China

**Keywords:** mulberry fruit, anthocyanin stability, acylation, antioxidant activity

## Abstract

**Research background:**

Anthocyanins possess valuable health-promoting activities with significant health benefits for humans. However, their instability is a limiting factor for their usage in functional foods and beverages.

**Experimental approach:**

In this work, a new method to enhance the stability of anthocyanins from mulberry fruit through acylation by using succinic acid as a selected acyl donor was explored. The Box-Behnken design of response surface methodology was applied to determine the optimized conditions for the acylation process.

**Results and conclusions:**

The highest acylation conversion rate was 79.04% at anthocyanins to succinic acid mass ratio 1:8.96, acylation duration 3 h and temperature 50 °C. Structural analysis of acylated anthocyanins revealed that succinic acid introduces a C-O-C bond and a hydroxyl group. The thermostability and light stability of mulberry anthocyanins were significantly improved after acylation, and the antioxidant activity expressed as total reducing power and Fe^2+^-chelating capacity of the acylated anthocyanins was also enhanced.

**Novelty and scientific contribution:**

Succinic acid acylation provides a novel method for stabilizing mulberry anthocyanins, as evidenced by the increased stability and antioxidant ability of anthocyanins, and thus facilitates its use in the food and nutraceutical industries.

## INTRODUCTION

Mulberry is a rich dietary source of different nutrients, such as alkaloids, flavonoids and polyphenols (*1*). Anthocyanins, a group of flavonoids, possess valuable health-promoting activities with significant health benefits for humans (*2*), and are believed to help prevent heart disease, and have antioxidant, antibacterial, anti-inflammatory and anticancer activities (*3*-*5*). Anthocyanins, with a C6-C3-C6 skeleton structure, generally occur in plants as glycosides and acylglycosides of anthocyanidins (aglycone) and differ from one another in the position of substitution of hydroxyl and methoxy groups in the β-ring (*6*). HPLC/ESI/MS analysis of mulberry fruits revealed the presence of four anthocyanins recognized as cyanidin 3-*O*-glucoside (C3G), pelargonidin 3-*O*-glucoside (P3G), cyanidin 3-*O*-rutinoside (C3R) and pelargonidin 3-*O*-rutinoside (P3R) (*7*). C3G and C3R are the major anthocyanins detected in mulberry fruit (*8*).

However, anthocyanins are extremely unstable and their composition is influenced by several factors during processing. Temperature plays an important role in the stability of anthocyanins. It has been suggested that anthocyanins exist in the chalcone structure. The anthocyanins become slightly unstable and turn colourless when the temperature rises to 60 °C (*9*). Another important factor that has been observed to have a strong influence on the stability of anthocyanins is pH value. Rein and Heinonen (*10*) demonstrated that there were four interconversion forms of anthocyanin in aqueous solution and different pH values lead to the different colours of solution. Besides these, light (*11*), metal ions (*12*), sugar content (*13*) and hydrogen peroxide (*14*) are also found to affect the stability of anthocyanins.

The relatively poor chemical stability of anthocyanins *in vitro* as well as *in vivo* (in plant cells or in the digestive tracts of animals) is a critical drawback and primary barrier that limits their wide and eﬀective applications, raising up concerns of the importance of reducing their degradation (*15*, *16*). To improve the anthocyanin stability, several modification methods have been studied, including glycosyl acylation (*15*), glycosylation (*16*), microencapsulation (*17*), metal chelation (*18*), liposomes (*19*) and Fe_3_O_4_ magnetic nanoparticle (*20*). From the nutritional viewpoint, acylated anthocyanins have been reported to possess antioxidant activity (*21*). Studies have shown that the presence of anthocyanins is different from plant to plant. To date, no information is available in the literature on the acylation of anthocyanins from mulberry fruit.

To predict the quality changes of anthocyanins during storage and processing, an acyl donor was selected for the acylation of mulberry anthocyanins in this study. A Box–Behnken design of response surface methodology was conducted for optimization of the acylation conditions. The thermostability and light stability of acylated anthocyanins were evaluated, and the antioxidant activity effects of acylated anthocyanins were demonstrated *in vitro*.

## MATERIALS AND METHODS

### Materials

Mulberry fruits collected from the plantation of the National Mulberry Orchard (Zhenjiang, PR China) were freeze-dried (EYELA FDU-2100; Tokyo Rikakikai Co., Ltd. Tokyo, Japan) and ground to powder. The cyanidin 3-*O*-glucoside standard was purchased from Putian Genesis Biotechnology Co., Ltd (Beijing, PR China). Succinic acid, l-malic acid, oxalic acid, pyridine, formic acid and acetonitrile were purchased from Sangon Biotech Co., Ltd (Shanghai, PR China). All other reagents used were of analytical grade.

### Mulberry anthocyanin extraction

Mulberry anthocyanins were extracted using acidified ethanol (*V*(ethanol):*V*(1.0 M HCl) 85:15, pH=1), assisted by ultrasound (*22*). The partially purified extracts by active macroporous resin D-101 were evaporated to dryness at 50 °C using a rotating evaporator (EYELA N1001; Tokyo Rikakikai Co., Ltd.), and were re-dissolved in acidified ethanol. Individual anthocyanins were separated, dried by freeze-drier (EYELA FDU-2100), and quantified using high-performance liquid chromatography (HPLC) system (Agilent Technologies Inc., Santa Clara, CA, USA). Anthocyanin structure was determined by Fourier transform infrared spectroscopy (FTIR; Varian Medical Systems, Palo Alto, CA, USA) for scanning in the absorption spectrum range.

### Preparation of anthocyanin glycosyl acylation

Acyl donors, including malic, succinic and oxalic acids, were selected for anthocyanin acylation by the method of Xu *et al.* (*23*). The reaction system was mixed with 2 mL pyridine as catalyst and 5 mg dried mulberry anthocyanins in 40 mL of 50% ethanol solvent and incubated at 50 °C for 4 h. Acylated anthocyanins were then evaporated at 40 °C, using a rotating evaporator (EYELA N1001; Tokyo Rikakikai Co., Ltd.) to remove the pyridine and ethanol, and dried using a vacuum freeze dryer.

### Optimization of anthocyanin acylation conditions

The following three-step procedure was used to optimize the anthocyanin acylation indices: (*i*) Plackett-Burman design to screen the four most influential factors that are known to affect anthocyanin acylation; (*ii*) acyl donors (malic, succinic and oxalic acids), the ratio of anthocyanin to donor (1:4, 1:8, 1:12, 1:16 and 1:20 mg/g), temperature (30, 40, 50, 60 and 70 °C) and reaction time (3.0, 3.5, 4.0, 4.5 and 5.0 h) were screened as single factors to determine the level of each factor that significantly improves acylated anthocyanin production; and (*iii*) Box-Behnken design to optimize the conditions for anthocyanin acylation using response surface methodology. Three virtual variables were used to estimate standard error. Based on the results obtained using the Plackett-Burman design, the three most influential factors that cover a wide range were selected to estimate the variation in anthocyanin acylation (*22*).

### Structural characterization of anthocyanins

The absorption spectra were recorded using a UV-Vis spectrophotometer (UV-2450; Shimadzu, Kyoto, Japan), scanning the spectrum range from 200 to 600 nm. The separated absorption spectra were obtained for non- and acylated anthocyanins. The structures of the acylated anthocyanins were characterized by scanning the infrared absorption spectrum range from 4000 to 400 cm^−1^ using FTIR spectroscopy.

### Calculation of conversion yield

The acylated anthocyanin conversion yield was calculated as previously described (*24*):



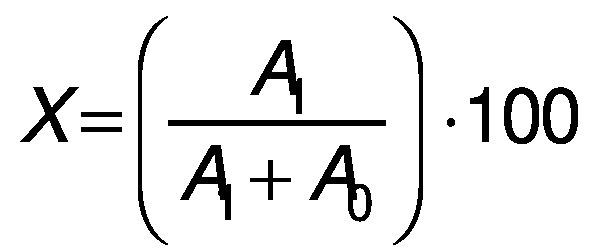



where *X* is the acylated anthocyanin conversion yield (%), *A*_0_ is the nonacylated anthocyanin peak area, and *A*_1_ is the acylated anthocyanin peak area.

### Stability tests of mulberry anthocyanins

#### Thermostability test

The thermostability of acylated anthocyanins was evaluated using 0.6 mg/mL of purified anthocyanins dissolved in 0.01% HCl solution (pH=1). The eﬀect of temperature on the anthocyanin content was investigated in a water bath at 70, 80 and 90 °C for 10 h in the dark in each treatment. Anthocyanin content was determined by measuring the absorbance at 520 nm using a UV-2450 UV-Vis spectrophotometer (Shimadzu, Kyoto). Anthocyanin retention rate (*R*) was calculated as follows (*25*):

*R*=(*A*_t_/*A*_0_)·100 /2/

where *A*_0_ represents the absorbance at the start of heating (*t*=0) and *A*_t_ represents the absorbance at time *t*.

#### Light stability test

To evaluate the light stability of the acylated anthocyanins, the anthocyanin solution as described above (0.6 mL) was placed in open glass cuvettes under 450 W light at 20 °C. After the exposure to light for 2, 4, 6, 8, 10 and 12 days, the absorption of solutions was measured and the anthocyanin retention rate was determined using Eq. 2.

### Antioxidant activity

Antioxidant activity was represented by the DPPH˙ free radical scavenging capacity, total reducing power, and Fe^2+^-chelating capacity, assayed according to Wu *et al.* (*26*).

#### DPPH free radical scavenging capacity

A volume of 2 mL of freshly prepared DPPH solution (1 mmol/L) was added to the anthocyanin extracts. The mixture was kept in the dark at 25 °C for 30 min and the sample absorbance was measured at 517 nm with a spectrophotometer (V-1800; Shimadzu Corp., Tokyo), using Trolox as the reference.

#### Fe(II) ion chelating capacity

A volume of 0.05 mL 2 mM FeCl_2_ solution and 0.1 mL 5 mM ferrozine solution were added to 3 mL anthocyanin extracts. The mixture was then shaken vigorously and incubated at room temperature for 10 min, and sample absorbance was measured at 562 nm with a spectrophotometer (V-1800; Shimadzu Corp., Tokyo).

#### Total reducing power

A volume of 2 mL of anthocyanin extracts was added to 2 μL of 1% potassium hexacyanoferrate(III). After mixing and incubation at 50 °C for 20 min, 2 mL of trichloroacetic acid were added. A volume of 2.5 mL of the supernatant was mixed with 2.5 mL of distilled water and 0.5 mL of FeCl_3_. After incubating the mixture for 10 min, the sample absorbance was measured at 700 nm with a spectrophotometer (V-1800; Shimadzu Corp., Tokyo).

### Statistical analysis

All of the analyses in this study were replicated thrice, and the results were expressed as mean values. Design-Expert v. 8.6 (*27*) and GraphPad Prism v. 7 (*28*) were used for experimental design and data analysis.

## RESULTS AND DISCUSSION

### Spectral analysis of acylated and nonacylated anthocyanins

**Fig. 1:**
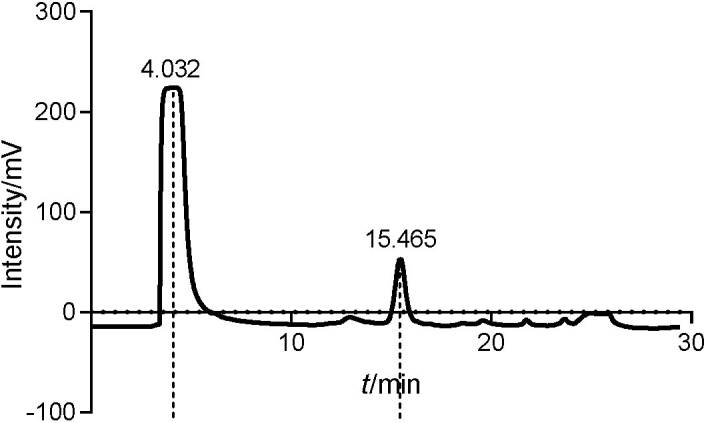
HPLC analysis of acylated (*t*=4.032 min) and nonacylated (*t*=15.465 min) anthocyanins

**Fig. 2:**
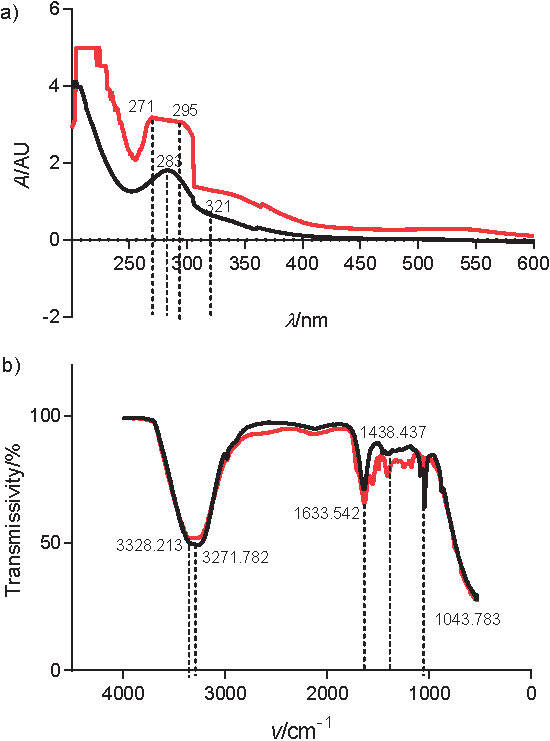
UV-visible spectra and FTIR characterization of acylated (red line) and nonacylated (black line) anthocyanins: a) UV-Vis spectra and b) infrared spectra

The acylated and nonacylated anthocyanins were detected by HPLC at 4.032 and 15.465 min, respectively (Fig. 1). The conversion yield of acylated anthocyanins was calculated according to the peak area. To examine the changes in the molecular structure, UV-Vis spectrophotometry was used to scan the absorption in the range from 200 to 600 nm. Fig. 2a shows that there are absorption peaks at 321 and 295 nm of nonacylated and acylated anthocyanins, respectively, indicating that the structure of the acylated anthocyanins is significantly different. The increase in the absorbance of acylated anthocyanins indicates that the acyl group in the anthocyanins is induced by succinic acid. It may be assumed that the acylation of anthocyanins was realized by introducing the acyl group, whereas their methylation was realized by introducing the methyl group (*29*).

Infrared spectrometry was performed by scanning in the range from 4000 to 400 cm^−1^ (Fig. 2b). The absorption peaks of nonacylated and acylated anthocyanins differed significantly in location with fluctuations in three regions. The strongest absorption peak is located in the first region from 3271 to 3328 cm^-1^. It corresponded to hydroxide radical stretching vibration peaks of aromatic ring and glycosyl in the structure. The absorption peaks at 1633 and 1438 cm^-1^ corresponded to the aromatic and heterocyclic ring skeletal vibration in the anthocyanins. The stretching vibration of acylated anthocyanins is enhanced relative to that of nonacylated anthocyanins. The third region is located from 1438 to 1043 cm^−1^, which is the characteristic absorption peak region in acylated anthocyanins. The stretching vibration region of the C-O-C group is located at 1250 cm^−1^, proving that acylation with succinic acid introduced the C-O-C and hydroxyl group.

In the case of methylation, the stretching vibration region of the C-H bond is located at 1395 cm^−1^, proving that it introduced the C-H bond and methyl group (*29*). Giusti *et al*. (*30*) reported that anthocyanin stability was enhanced signiﬁcantly through hydrophobic and ’π−π’ interactions. It could be concluded that based on the modification method, appropriate changes are induced on the relative chemical group leading to enhanced anthocyanin stability.

### Optimization of anthocyanin acylation

Based on a previous study (*31*), three potential influencing factors, the ratio of anthocyanin to donor, temperature and reaction time, were studied to optimize the anthocyanin acylation. The single-factor tests to assess anthocyanin conversion rate as depicted in Table 1 show that the acyl donor is succinic acid, anthocyanin to donor mass ratio is 1:8, acylation time is 3.5 h and acylation temperature is 40 °C.

**Table 1:** Selection of potential explanatory factors (*) for anthocyanin acylation

Acyl donor	Conversion rate/%	*m*(anthocyanin):*m*(donor)	Conversion rate/%
Succinic acid	(71.5±3.4)*	1:4	60.0±4.0
Malic acid	65.00±2.066	1:8	(71.6±3.9)*
Oxalic acid	23.50±2.905	1:12	54.97±4.565
		1:16	45.60±3.804
		1:20	39.73±3.400
Temperature(acylation)/°C	Conversion rate/%	*t*(acylation)/h	Conversion rate/%
30	64.37±3.80	3.0	64.47±3.232
40	(70.43±2.36)*	3.5	(72.80±2.551)*
50	62.87±2.631	4.0	62.70±2.352
60	57.47±2.386	4.5	59.73±3.272
70	48.43±3.009	5.0	52.57±2.802

The conversion rate for different mass ratio of anthocyanin to acyl donor was measured under different acylation temperatures, and conversion rate at different acylation time was measured using succinic acid as donor

Response surface analysis was adapted to optimize the process conditions of anthocyanin acylation. The results of the regression analysis are shown in Table 2. Using multiple regression analysis, the results were fitted to a second-order polynomial equation. The regression equation between *Y* and A, B and C was established as follows:

*Y*=72.6-2.55A+2.05B+3.35C-5.60AB+8.90AC-15.90BC-24.85A^2^-2.65B^2^-9.55C^2^ /3/

**Table 2:** ANOVA results for the regression response surface model

Source	Sum of squares	df	Mean square	F value	p-value Prob>F
Model	4814.92	9	534.99	13.53	0.0012**
A	52.02	1	52.02	1.32	0.2891
B	33.62	1	33.62	0.85	0.3872
C	89.78	1	89.78	2.27	0.1756
AB	125.44	1	125.44	3.17	0.1181
AC	316.84	1	316.84	8.01	0.0254*
BC	1011.24	1	1011.24	25.58	0.0015**
A^2^	2600.09	1	2600.09	65.76	0.0001**
B^2^	29.57	1	29.57	0.75	0.4158
C^2^	384.01	1	384.01	9.71	0.0169*
Residual	276.78	7	39.54		
Lack-of-fit	188.22	3	62.74	2.83	0.1701
Pure error	88.56	4	22.14		
Cor total	5091.70	16			
R^2^	0.9456				
Adj-R^2^	0.8758				

* p<0.05, **p<0.01

To understand the significance of the linear relationship between the response and independent variables, ANOVA was performed for the regression response surface model. Table 2 shows that the model accounted for 94.56% variability in the response, indicating that it is a good fit, which was confirmed by the calibration coefficient value, R=0.9456. The non-significance of the lack-of-fit parameter shows that the quadratic regression equation is a good estimator of the response. The “Prob>F” value was less than 0.001 (Table 2), indicating that the model is significant. This suggests that the most critical independent factors were the mass ratio (A), acylation time (B), and acylation temperature (C), in that order.

**Fig. 3:**
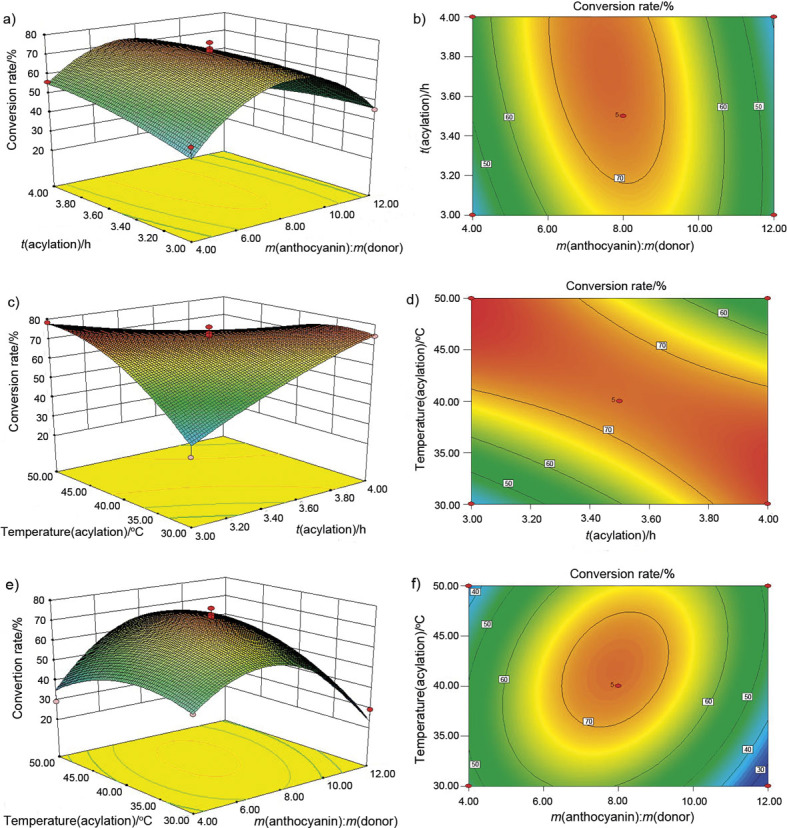
Response surface and contour plots for anthocyanin acylation: a and b) interaction of mass ratio and acylation time, c and d) interaction of acylation temperature and mass ratio, and e and f) interaction of acylation temperature and acylation time

To determine the optimal acylation conditions, graphs were plotted to compare the influential parameters (Fig. 3). The response surface graphs, each relating two out of the three factors, consist of ellipses with one centre, regardless of their orientation; the edges of the surfaces formed vaults with peaks. The optimum acylation conditions using succinic acid were as follows: anthocyanins to succinic acid mass ratio 1:8.96, acylation time 3 h and acylation temperature 50 °C. Under these conditions, the predicted conversion yield was 79.04%, and the validation value 80.7%, indicating its high reliability.

### Thermostability of acylated anthocyanins

**Fig. 4:**
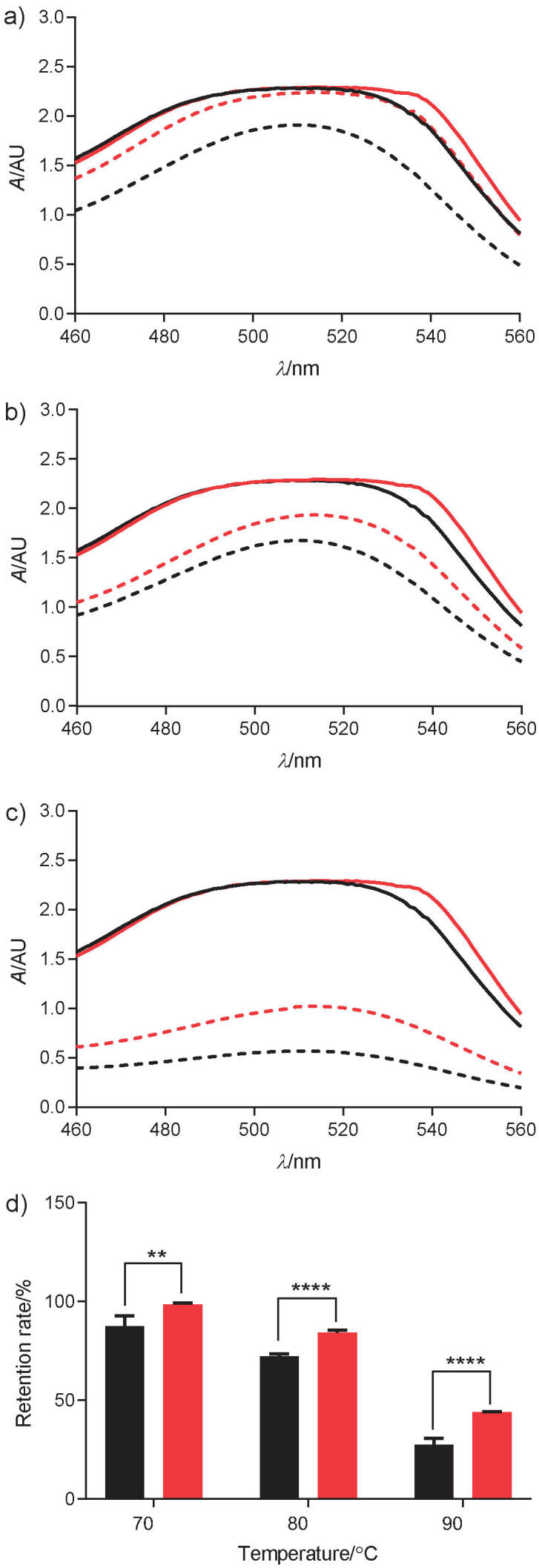
Thermostability of acylated and nonacylated anthocyanins measured spectrophotometrically in darkness for 10 h at different temperatures: a) 70 °C, b) 80 °C, c) 90 °C, and d) retention rate of acylated (red) and nonacylated anthocyanins (black) at different temperatures. Full line: acylated (red) and nonacylated (black) anthocyanins without thermal stimulation; dashed line: acylated (red) and nonacylated (black) anthocyanins with thermal stimulation for 10 h

Thermostability of acylated anthocyanins was evaluated following storage at different temperatures for 10 h in darkness. The absorbance of nonacylated anthocyanins from 460 to 560 nm at 70 (Fig. 4a), 80 (Fig. 4b) and 90 °C (Fig. 4c) showed the same trend of acylated anthocyanins lower than that of nonacylated anthocyanins. The retention rates of acylated anthocyanins were 98.41, 84.25 and 44.10%, and those of nonacylated anthocyanins were 87.49, 72.15 and 27.44%, respectively at 70, 80 and 90 °C (Fig. 4d). This indicates that the acylated anthocyanins were relatively more stable than nonacylated anthocyanins at high temperatures, and that mulberry fruit anthocyanins can be preserved well at these temperatures.

Temperature and light degrade anthocyanin stability significantly and reduce biological activities (*32*). Thermal treatment is believed to break down anthocyanins or their conjugated sugars into small molecules like aldehydes, benzoic acid derivatives or synonymous anthocyanidins (*33*, *34*). In this study, the anthocyanin retention rate decreased sharply from 87.49% at 70 °C to 27.44% at 90 °C after 10 h. However, the retention rate of acylated anthocyanins decreased from 98.41% at 70 °C to 44.10% at 90 °C. These results are consistent with those of previous studies (*16*, *24*, *35*).

### Light stability of acylated anthocyanins

**Fig. 5:**
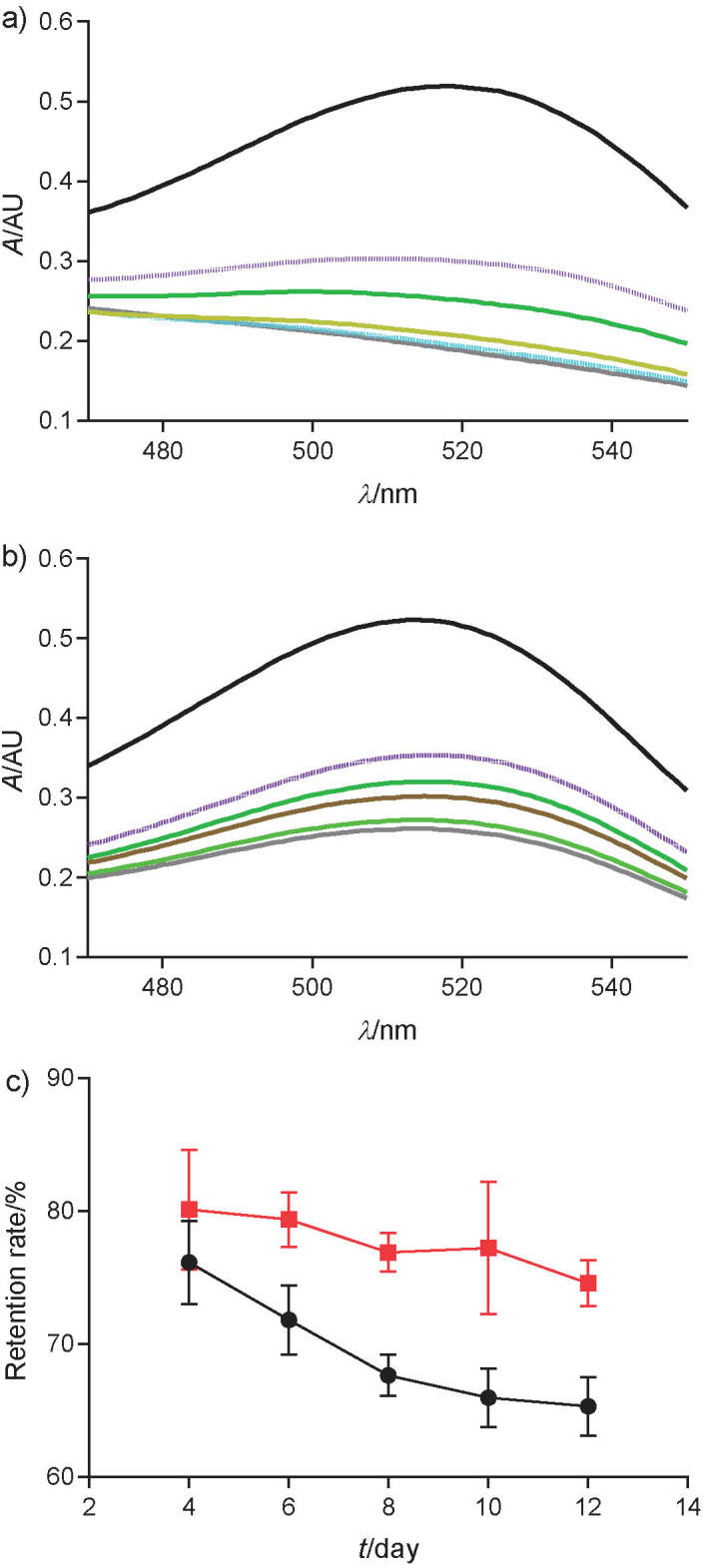
Light stability of: a) nonacylated and b) acylated anthocyanins when exposed to 400 W light: colour lines from up to down indicate 0, 4, 6, 8, 10 and 12 day, respectively, and c) retention rate of nonacylated (black line) and acylated (red line) anthocyanins exposed to 400 W light for different durations

To investigate the effect of acylation on the light stability of acylated anthocyanins, anthocyanin retention rate was measured after exposure to 400 W light for 2, 4, 6, 8, 10 and 12 days at 20 °C. The absorbance of anthocyanins (Fig. 5a) and acylated anthocyanins (Fig. 5b) from 460 to 560 nm was observed to significantly decrease in proportion to the duration of exposure to 400 W light. The anthocyanin retention rate decreased steeply from 76.13 to 65.97% during 10 days (Fig. 5c). However, the acylated anthocyanin retention rate was 80.10% for 4 days and 77.21% for 10 days, without significant difference. It indicates that the light stability of acylated anthocyanins is much better than that of methylated anthocyanins (*16*).

In terms of light stability, acylated anthocyanins synthesized by lipase-catalysed transesteriﬁcation were more stable than their nonacylated glucosides under illumination with white ﬂuorescent light (*36*). Several studies have reported the potential ability of acyl groups to donate electrons to anthocyanins (*37*, *38*), enhancing the stability of acylated anthocyanins under light irradiation.

### Antioxidant activity of acylated anthocyanins

**Fig. 6:**
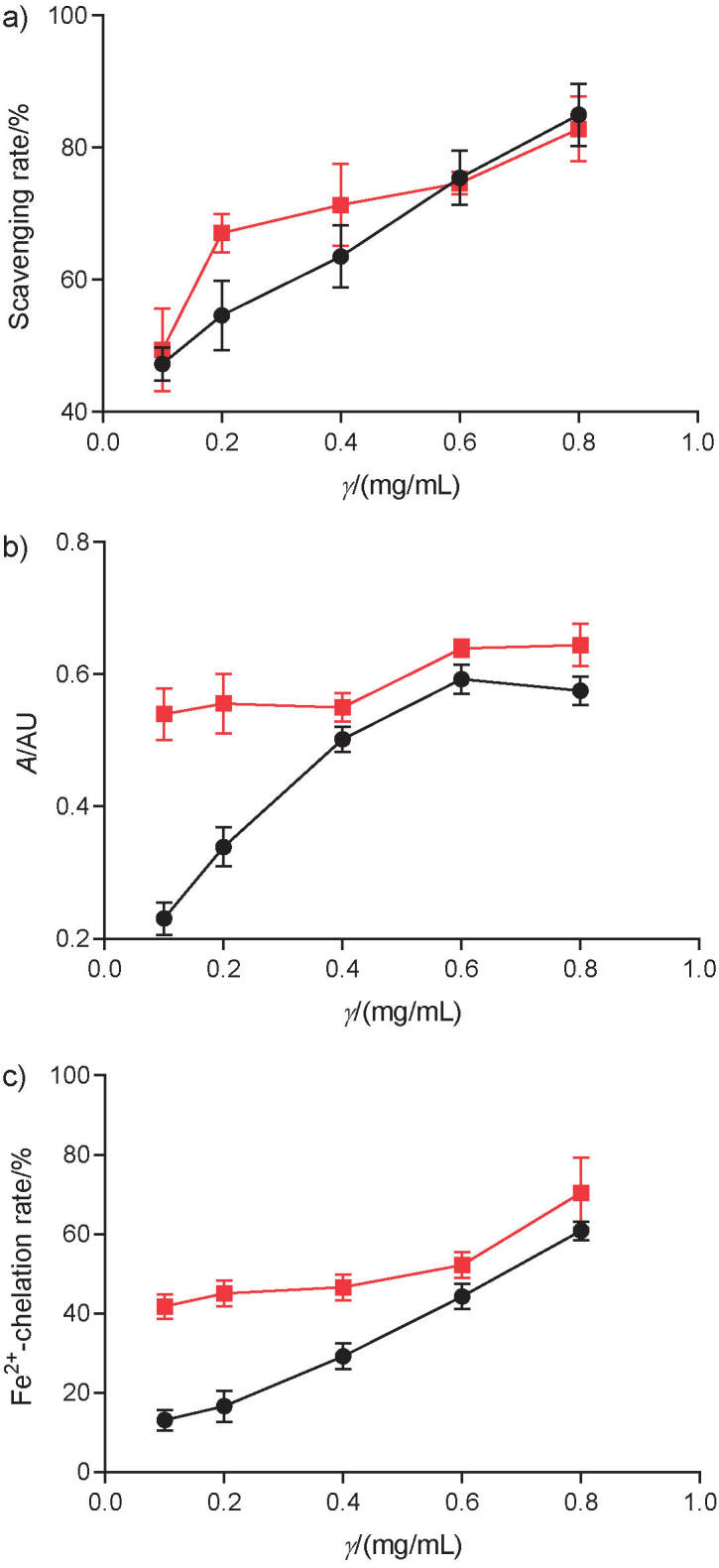
Antioxidant ability of acylated (red line) and nonacylated (black line) anthocyanins: a) DPPH free radical scavenging, b) total reducing power and c) Fe^2+^-chelating capacity.

The antioxidant properties that were assessed *in vitro* were DPPH radical scavenging, total reducing power and Fe^2+^-chelating capacity (Fig. 6). Nonacylated and acylated anthocyanins exhibited these properties in a concentration-dependent manner. DPPH radical scavenging activity was similar in the two groups, without significant differences (Fig. 6a). However, total reducing power and Fe^2+^-chelating capacity were detected as increasing rapidly with increasing concentration and being significantly higher in acylated anthocyanins. At 0.8 mg/mL Fe^2+^, nonacylated and acylated anthocyanins reached levels of 62.2 and 70.5%, respectively (Fig. 6c).

Anthocyanin acylation using succinic acid as a donor significantly enhanced the retention rate of anthocyanins at a certain degree of temperature and light conditions and improved their antioxidant activities in terms of total reducing power and Fe^2+^-chelating capacity. The present result is in line with those of our previous reports (*16*). However, the exact mechanisms whereby acylation enhances anthocyanin stability need to be explored further.

## CONCLUSIONS

In this study, succinic acid was selected as an acyl donor for anthocyanin acylation to improve anthocyanin stability. The optimized acylation conditions were as follows: anthocyanin to succinic acid mass ratio 1:8.96, acylation time 3 h and acylation temperature 50 °C. The acylation of anthocyanins by succinic acid may be attributed to the formation of a C-O-C bond and a hydroxyl group. Our thermostability and light stability results show that acylation significantly enhances the preservation rate of anthocyanins and improves their antioxidant activity. In the case of the light stability, the acylation offered better results than the methylation of anthocyanins. Thus, acylation of anthocyanins may be a novel method for stabilizing anthocyanins in mulberry fruit enabling their use as a commercial food ingredient.
